# Molecular Characterization and Antimicrobial Resistance Evaluation of *Listeria monocytogenes* Strains from Food and Human Samples

**DOI:** 10.3390/pathogens14030294

**Published:** 2025-03-18

**Authors:** Annamaria Castello, Vincenzina Alio, Marina Torresi, Gabriella Centorotola, Alexandra Chiaverini, Francesco Pomilio, Ignazio Arrigo, Anna Giammanco, Teresa Fasciana, Marco Francesco Ortoffi, Antonietta Gattuso, Giuseppa Oliveri, Cinzia Cardamone, Antonella Costa

**Affiliations:** 1 IZSSI—Istituto Zooprofilattico Sperimentale della Sicilia “A. Mirri”, Via Gino Marinuzzi 3, 90129 Palermo, Italy; vincenzina.alio@izssicilia.it (V.A.); giuseppa.oliveri@izssicilia.it (G.O.); cinzia.cardamone@izssicilia.it (C.C.); antonella.costa@izssicilia.it (A.C.); 2 IZSAM—Istituto Zooprofilattico Sperimentale dell’Abruzzo e del Molise G. Caporale, Via Campo Boario, 64100 Teramo, Italy; m.torresi@izs.it (M.T.); g.centorotola@izs.it (G.C.); a.chiaverini@izs.it (A.C.); f.pomilio@izs.it (F.P.); 3 Dipartimento di Promozione della Salute, Materno-Infantile, di Medicina Interna e Specialistica di Eccellenza “G. D’Alessandro”, University of Palermo, 90127 Palermo, Italy; ignazio.arrigo90@gmail.com (I.A.); anna.giammanco@unipa.it (A.G.); teresa.fasciana@unipa.it (T.F.); 4 Dipartimento di Sicurezza Alimentare, Nutrizione e Sanità Pubblica Veterinaria—Istituto Superiore di Sanità, 00161 Roma, Italy; marco.ortoffi@iss.it (M.F.O.); antonietta.gattuso@iss.it (A.G.)

**Keywords:** *Listeria monocytogenes*, food, food production environment, antimicrobial resistance, serotype, MLST

## Abstract

*Listeria monocytogenes* is an important foodborne pathogen, markedly persistent even in harsh environments and responsible for high hospitalization and mortality rates. The aim of the present study was to detect the strains circulating in Sicily over a five-year period and characterize their antimicrobial resistance profiles. The key element of this study was the sharing of data among various entities involved in food control and clinical surveillance of listeriosis in order to develop an integrated approach for this pathogen. A total of 128 isolates were analyzed, including 87 food-source strains and 41 clinical specimens. Whole-genome sequencing (WGS) was performed for sequence type (ST) and clonal complex (CC) identification through multilocus sequence typing (MLST) analysis. Antimicrobial resistance was assessed using the Kirby–Bauer method. The majority of strains belonged to serotype IVb (34/41 and 53/87 of clinical and food-source isolates, respectively) and were subtyped as CC2-ST2 (28/34 and 41/53 of clinical and food-source isolates respectively). Most of the isolates were susceptible to the main antimicrobials recommended for treatment of listeriosis. Resistance (R) and intermediate resistance (I) percentages worthy of attention were found against oxacillin (R: 85.9%) and clindamycin (I: 34.6%) in the food-source isolates and trimethoprim/sulfamethoxazole (R: 29.23%) in the clinical isolates. Also, 7.7% of the food-source isolates were multidrug resistant. Our results highlight how the punctual comparison between food and clinical strains is an essential tool for effectively tracking and preventing foodborne outbreaks.

## 1. Introduction

*Listeria monocytogenes* is a Gram-positive, non-spore-forming, and motile pathogenic bacillus. The capacity to grow and survive in a wide range of temperatures (from below 0 °C to 44 °C) and pH conditions (pH 4.2–9.5) [1], as well as at high salt concentrations (10%, *wt*/*vol*) [2], make *Listeria monocytogenes* capable of colonizing various environments, included food production and processing plants. Moreover, its ability to form biofilm makes this bacterium resistant to disinfectants and able to persist on surfaces for years or even decades [3,4]. Disease manifestations can include severe clinical pictures and high mortality rates, especially in frail individuals such as the elderly, pregnant women, infants, and immunocompromised adults. In Western countries, listeriosis is an increasingly worrying public health issue. In fact, according to the latest report released by the EFSA (European Food Safety Authority) and ECDC (European Centre for Disease Prevention and Control), listeriosis ranks fifth among the most common zoonoses in the EU, with the highest notification rate and number of cases reported since 2007, when surveillance at the EU level began. Listeriosis is also confirmed as one of the zoonoses under clinical surveillance characterized by the worst outcomes due to the high rate of hospitalizations and elevated morbidity and mortality [5].

Most listeriosis cases appear to be sporadic, and reported outbreaks are usually small [3]. Nevertheless, the use of improved molecular subtyping methods for cluster analysis suggests that outbreaks may involve more human cases than previously assumed [2]. Indeed, since the application of enhanced foodborne pathogen subtyping procedures, a large number of human listeriosis outbreaks have been reported in various countries, included multi-country and prolonged multi-country outbreaks [2,6]. Finally, as reported by EFSA, it is difficult to establish causative links between human cases and foods. Results from studies attributing human cases to different animal sources are limited, as well as source attribution in general, due to both low representativeness of isolates from all relevant sources and difficulties in identifying their origin [3]. The use of NGS techniques has proven to be a potentially beneficial approach for both improved outbreak detection and stronger source attribution [3]. With the ageing of the European population [7] and the increase in chronic age-related diseases [8], more people are entering the high-risk categories for severe listeriosis. *L. monocytogenes* is a multifaceted pathogen that not only represents a serious threat to human health and a challenge to food safety but also plays a relevant pathogenic role among farm animals, especially ruminants [9]. Wild mammals [10] and even birds [11,12,13] can act as reservoirs and sources of infection, introducing this bacterium into the fecal–oral transmission circuit and causing its spread even in environments not strictly associated with food production and processing. In fact, according to references, *L. monocytogenes* can be isolated from irrigation water, agricultural soil [14], and even the urban environment [15]. For these reasons, more attention to the prevention and control of the disease is needed, as well as improvements in outbreak detection, prompt source attribution, and tracking. The implementation of techniques for strain detection, typing, and cross-sectoral collaboration, including sequence data-sharing, is essential for improved surveillance of listeriosis [16]. This paper describes data obtained as part of a 4-year pilot study aimed at implementing an integrated medical–veterinary system for the surveillance of listeriosis in Sicily, in accordance with requests from both the Ministry of Health of Italy and the European Union (EU).

## 2. Materials and Methods

**Figure 1:**
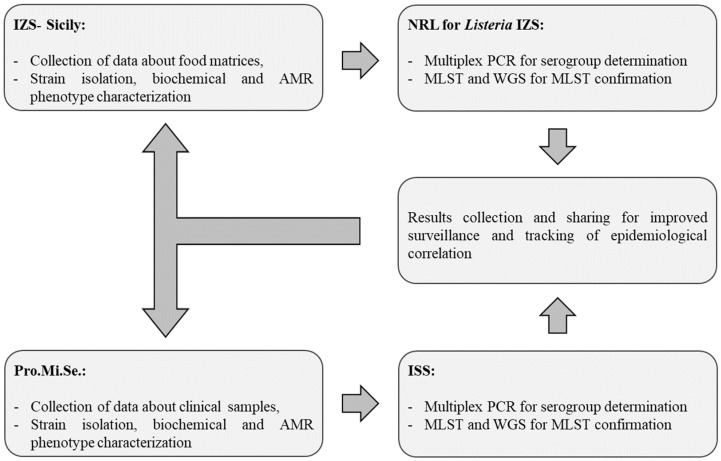
Flowchart depicting the technical–scientific methodologies applied for the detection and characterization of strains and the approach of data management and sharing applied to implement and improve an integrated system for the surveillance of listeriosis. IZS—Istituto Zooprofilattico Sperimentale; AMR—Antimicrobial resistance; NRL—National Reference Laboratory; MLST—Multilocus Sequence Typing; WGS—Whole Genome Sequencing; Pro.Mi.Se.—Dipartimento di Promozione della Salute, Materno-Infantile, di Medicina Interna e Specialistica di Eccellenza.

This study was developed by IZS (Istituto Zooprofilattico Sperimentale) of Sicily, in collaboration with Pro.Mi.Se. (Department of Health Promotion, Mother and Child Care, Internal Medicine and Medical Specialties, Policlinico “P. Giaccone” hospital in Palermo); the National Reference Laboratory (NRL) for *Listeria monocytogenes* of IZS Abruzzo e Molise “G. Caporale” (https://www.izs.it/IZS/National_Activities/National_Reference_Laboratories_1/Listeria_monocytogenes (accessed on 26 February 2025)), and the Department of Food Safety, Nutrition and Veterinary Public Health of ISS (Istituto Superiore di Sanità). The flowchart given below (Figure 1) depicts the technical–scientific methodologies applied for the detection and characterization of strains and the approach of data management and sharing applied to implement and improve an integrated system for the surveillance of listeriosis.

The strains isolated from food at IZS, together with the related metadata (such as isolation source, sampling site, identification tests carried out), were sent to the NRL for *L. monocytogenes* to be characterized with sequencing using the NextSeq 500 Illumina platform (Illumina, San Diego, CA, USA). The results were analyzed with the bioinformatics and data collection platform GenPat. The strains isolated at Pro.Mi.Se., together with the related epidemiological data, were sent to the ISS for molecular characterization using whole-genome sequencing (WGS). Sequencing was performed using the MySeqDx Illumina platform, and analysis was performed automatically using the bioinformatics and clinical strain data collection platform IRIDA (Integrated Rapid Infectious Disease Analysis) ARIES (Advanced Research Infrastructure for Experimentation in Genomics).

### 2.1. L. monocytogenes from Food Samples

This study concerns *L. monocytogenes* strains isolated from food matrices, collected in Sicily between 2020 and 2023. According to Reg. 2073/2005/EC, five sample units were tested for each sample, and one *L. monocytogenes* isolate was collected from each positive sample unit. The detection and isolation of *L. monocytogenes* were performed according to ISO 11290-1:2017, as follows: 25 g of sample was diluted 1:10 (*w*/*v*) in half Fraser broth (Oxoid, Milan, Italy), homogenized using a stomacher machine and incubated at 30 °C for 24 h (pre-enrichment). A 1:100 (*v*/*v*) dilution of the pre-enriched inoculum in 10 mL of Fraser broth (Oxoid, Milan, Italy) was incubated at 37 °C for 24 h. Finally, a loopful of the inoculum was streaked onto two selective media plates, ALOA (Agar Listeria according to Ottaviani and Agosti) and Oxford agar (Oxoid, Milan, Italy), and incubated at 37 °C for 48 h. Presumptive colonies were confirmed using confirmatory tests and miniaturized biochemical tests (API Listeria, BioMerieux, Marcy l’Etoile, France) [17].

The antimicrobial susceptibility of *L. monocytogenes* isolates was assessed based on a subsample of 78 isolates using the Kirby–Bauer disk diffusion method, as recommended by the European Committee on Antimicrobial Susceptibility Testing (EUCAST). Briefly, colonies were suspended in 1 mL of 0.9% NaCl solution, and the turbidity of this suspension was adjusted to a 0.5 McFarland standard. The suspension was used to inoculate Mueller–Hinton-F agar (Oxoid, Milan, Italy). The following 16 antimicrobials were used: penicillin G (P; 10 U/disc), ampicillin (Amp; 10 μg/disc), amoxicillin–clavulanic acid (Amc; 20 μg–10 μg/disc), oxacillin (Oxa; 1 µg/disc), cephalothin (Kf; 30 μg/disc), imipenem (Imp; 10 μg/disc), clindamycin (Da; 30 µg/disc), chloramphenicol (C; 30 μg/disc), erythromycin (E; 15 μg/disc), ciprofloxacin (Cip; 5 μg/disc), gentamicin (Cn; 10 μg/disc), kanamycin (K; 30 μg/disc), streptomycin (S; 10 μg/disc), trimethoprim/sulfamethoxazole (Sxt; 25 μg/disc), tetracycline (Te; 30 μg/disc) and vancomycin (Va; 30 µg/disc). The diameters of the zones of inhibition surrounding each disk were measured using a digital caliper and interpreted according to the guidelines of EUCAST [18]. In cases where the EUCAST guidelines lacked resistance criteria for listeria, the CLSI guidelines recommended for *Staphylococcus aureus* and *Enterococcus* spp. were followed [19,20,21].

The strains were stored at −20 °C in Microbank (PRO-LAB diagnostics, Biolife Italiana S.r.l., Milan, Italy) and sent, together with the related metadata (such as isolation source, sampling site, identification tests carried out), to the NRL for *L. monocytogenes* for serogroup determination (multiplex PCR) [22] and further molecular investigation through whole genome sequencing (WGS).

### 2.2. L. monocytogenes from Clinical Samples

The strains of *L. monocytogenes* were isolated from the biological samples of patients with invasive listeriosis, hospitalized in different hospitals in Palermo from 2019 to 2023, according to the following indications.

Demographic data (age, gender, residence), clinical data (symptoms, clinical status, date of sample collection and hospital where the biological sample was collected, outcome if already available), risk factors (cancer, solid organ transplantation, inflammatory diseases, age > 65 years, pregnancy status), type of biological sample (blood culture, cerebrospinal fluid, ascitic fluid, nasal swab, placental swab, meconium), and antibiotic therapy were reported in a questionnaire provided by the ISS and associated with each clinical strain. In most cases, the outcome was unknown at the time of clinical strain shipment. Data were collected anonymously in accordance with patient privacy.

Blood samples were inoculated in bottles containing Plus Aerobic/F medium, an enriched soybean–casein digest broth with CO_2_, and an incorporated cation-exchange resin that is effective in removing β-lactam antibiotics, gentamicin/penicillin, and vancomycin. All bottles were incubated for 12 h at 35 °C with continuous agitation using a Bactec 9120 automated blood culture system (Becton Dickson Manifacture, Sparks, MD, USA). This is an incubator featuring fluorescent sensor technology for continuous monitoring of the quantity and rate of CO_2_ production, as these are indicative of microbial growth [23]. The samples that tested positive for microbial growth were examined using microscopy, mass spectrometry (MALDI-TOF, Bruker, Leipzig, Germany), molecular biology (BIOFIRE film array blood culture ID panel), and cultivated on 5% sheep blood agar medium. CSF samples were examined microscopically after Gram staining, analyzed using a molecular method using the BIOFIRE film array Meningitis/Encephalitis (ME) panel system (BioFire Diagnostics, Salt Lake City, UT, USA) and inoculated on 5% sheep blood agar medium. Ascitic fluid and nasal swabs were directly inoculated on 5% sheep blood agar medium for cultivation. All of the 5% sheep blood agar plates were incubated at 37 °C for 24 h in aerobic conditions.

After growth, the suspected colonies were subcultivated on a 5% sheep blood agar medium and identified using matrix-assisted laser desorption/ionization–time of flight (MALDI-TOF) mass spectrometry (Bruker, Leipzig, Germany). The antimicrobial susceptibility of the *L. monocytogenes* isolates was assessed using the Kirby–Bauer method (disc diffusion technique). The following antimicrobials were used: ampicillin (10 µg/disc), erythromycin (15 µg/disc), meropenem (10 µg/disc), penicillin G (10 µg/disc), and trimethoprim/sulfamethoxazole (25 µg/disc). The diameters of the zones of inhibition surrounding each disk were measured using a digital caliper and interpreted according to the guidelines of the European Committee on Antimicrobial Susceptibility Testing [18]. All of the isolates collected were evaluated for serogroup definition and stored at −20 ° C in Microbank. Finally, the isolates stocked in Microbank were sent to the ISS for further molecular analyses, together with the aforementioned demographic and clinical data related to the source patients.

### 2.3. Molecular Investigations, Bioinformatic and Cluster Analyses

Molecular investigations were performed as follows: the DNA extraction was performed using the QIAamp DNA Mini Kit (QIAGEN, Hilden, Germany) according to the manufacturer’s instructions. DNA was quantified using a Qubit ds DNA HS (High Sensitivity) Assay Kit (Invitrogen, Carlsbad, CA, USA), while the quality parameters of purity (A260/A239 and A260/280 ratios) and DNA integrity (DIN) were assessed based on Biospectrometer fluorescence and using an Agilent 2200 TapeStation system (Agilent Technologies, Santa Clara, CA, USA), respectively. The DNA was processed using multiplex PCR according to previous studies [22] as follows: amplification reactions were carried out at a final volume of 25 µL in a GeneAmp^®^ PCR systems 9700 thermal cycler (Applied Biosystem^®^, Carlsbad, CA, USA). The following conditions were applied: a first step of denaturation at 94 °C for 10 min and 35 cycles of a 3-step amplification (denaturation at 94 °C for 40 s, annealing at 53 °C for 90 s, and extension at 72 °C for 90 s), followed by a final extension step at 72 °C for 10 min. The PCR products were separated using capillary electrophoresis with the QIAxcel DNA Screening Kit system (Qiagen, Milan, Italy) and analyzed according to the dedicated software (Qiaxcel ScreenGel 2.0 software, Redwood City, CA, USA) provided by the manufacturer. The target genes for serogroup determination and the expected sizes of the amplification products are listed in Table 1.

Serogroup attribution was carried out according to Doumith et al. [26] and Kéruanton et al. [25] and successively confirmed in silico via WGS. Multilocus sequence typing (MLST) was performed according to Pasteur’s reference schemes (https://bigsdb.pasteur.fr/ (accessed on 28 February 2025)). WGS was carried out as previously reported by Centorotola et al. (2023) [27] using the Illumina platform. Referring to the sequences obtained from food source isolates, the WGS data analysis was performed using an accredited in-house pipeline (https://github.com/genpat-it/ngsmanager/ (accessed on 28 February 2025)), and quality was assessed according to ISO 23418:2022 [28].

Furthermore, referring to the isolates from food and clinical samples belonging to CC2-ST2, core genome multilocus sequence typing (cgMLST) was applied. This analysis was performed according to Pasteur’s reference schemes (https://bigsdb.pasteur.fr/ (accessed on 28 February 2025)), considering the threshold of ≤7 allelic distance (AD) for the cgMLST cluster definition [29]. The software GrapeTree (https://achtman-lab.github.io/GrapeTree/MSTree_holder.html (accessed on 28 February 2025)) [30] was used to obtain the Minimum Spanning tree (MSTreeV2). Single nucleotide polymorphism (SNP) analysis was performed using the CFSAN pipeline [31] using *L. monocytogenes*_11 (mapped against GCF_000195395.4) as the reference.

Moreover, the *L. monocytogenes* CC2-ST2 genomes were also characterized in silico using BIGSdb-*Lm* database tools (accessed on February 2025) for “Virulence”, “Antibiotic resistance”, “Metal and disinfectant resistance”, and “Stress Islands”. The *L. monocytogenes* CC2-ST2 genome assemblies were deposited in DDBJ/ENA/GenBank under the BioProject ID PRJNA1234508.

## 3. Results

A total of 128 strains of *L. monocytogenes* were isolated and characterized during the five-year period of 2019–2023. Among these, 87 strains were isolated from food and 41 strains were isolated from clinical samples.

### 3.1. L. monocytogenes from Food Samples

All the details related to the *L. monocytogenes* isolates from food are reported in Table 2.

Among the food samples, serogroup IVb was the most abundant (53/87, 69%), followed by serogroups IIa (25/87, 28.7%) and IIb (7/87, 7%), detected in 7/17 (41.2%) of the samples of fish products, and IIc (2/87, 2.3%), detected in 2/21 (9.5%) of the samples of meat and meat products. Overall, the MLST analysis identified 15 clonal complexes (CCs); in particular, CC2 (43/87, 49.4%), CC8 (7/87, 8%), CC1 (6/87, 6.9%), CC199 (5/87, 5.7%), CC426 and CC6 (4/87, 4.6% each), and CC121 and CC155 (3/87, 3.45% each).

The majority of the strains belonging to serogroup IVb (34/53, 64.1%) were detected in the dairy products sampled from the same production plant in 2020 and 2022. All of them belonged to CC2 and Sequence Type 2 (ST2), suggesting a condition of persistent contamination with *L. monocytogenes* in the producing plant.

Referring to the isolates from ricotta cream and ricotta cheese, those samples came from the same dairy plant, sampled at the production plant (ricotta cheese) and distribution site (ricotta cream). Both types of samples were found to be contaminated by two different strains of *L. monocytogenes*, namely serogroup IIa, CC199, ST199 (1/2 samples of ricotta cheese and 4/5 sampling units of ricotta cream), and serogroup IVb, CC6, ST6 (1/2 samples of ricotta cheese and 1/5 sampling units of ricotta cream). These data underline the possibility, for the same plant and consequently for the same foodstuff, of being contaminated by different strains, potentially characterized by different resistance and pathogenicity characteristics. Serogroup IVb was also detected in 8/21 (38.1%) samples of meat and meat products, 2/17 (11.8%) samples of fish products, and 5/5 (100%) samples of vegetables. Greater variability among the isolated strains was observed among the meat and meat products and fish products in terms of both serogroup and CC and ST (Table 2).

Referring to the assessment of antimicrobial susceptibility, the majority of the strains were resistant (R) to oxacillin (85.9%) and sensitive to the remaining antimicrobials, except for clindamycin (intermediate resistance—I: 34.6%). Few strains showed divergent resistance/intermediate resistance profiles compared to the majority phenotype; in particular, the n.1 strain from ricotta cream (R: OXA, DA, K, SXT), n.3 strains from stretched-curd cheese (n.1/3 R: OXA, I: CN, K and n.2/3 R: AMP, AMC, OXA, KF, CIP, CN, K, S, I: E), n.1 strain from meat and meat products (R: P, I: OXA), n.1 strain from seafood salad (R: P, OXA), n.4 strains from smoked salmon/tuna (n.2/4 R: P, OXA, K, VA, I: DA; n.1/4 R: P, K, VA, I: DA and n.1/4 R: P, I: OXA), and n.1 strain from vegetables (R: OXA, KF).

### 3.2. L. monocytogenes from Clinical Samples

The *L. monocytogenes* clinical strains were community-acquired. The isolates were obtained from 41 patients (individual cases) hospitalized in different hospitals in Palermo city, Sicily, between 2019 and 2023. According to demographic data, a greater number of cases was recorded among males compared to females (28 vs. 13), confirming the greater susceptibility of men reported in literature [32].

*L. monocytogenes* infections were reported mainly in the age group over 65 years (22/41 patients), eleven were under the age of 65 years, one was a three-year-old child, and seven were newborns who had acquired the infection at birth.

In addition to advanced age, which represents a known risk factor for listeriosis, many patients also had concomitant pathologies, including cancer, ulcerative rectocolitis, cardiac diseases, COVID-related lung diseases, and obesity. The most common clinical manifestation was bacteremia with or without sepsis, diagnosed in 25/41 patients (61%). Meningitis was diagnosed in 13/41 patients (32%), in association with sepsis and pleural effusion, in three cases and one case, respectively. Bacteremia was diagnosed in 12/41 patients (29%). Pregnancy-associated infections (8/41 cases) included listeriosis in newborns during the first month of life and maternal–fetal infections. All of the mothers hospitalized were affected by intrauterine infection; the outcomes of the pregnancy-associated infections were 5/8 preterm births and 1/8 stillbirth. All newborns had respiratory distress. In most cases (25/41, 61%), the isolation site was the blood, while in 13/41 (31.7%) of the cases, the isolation site was CSF (cerebrospinal fluid). Moreover, three cases were positive in the ascitic fluid, umbilical catheter, and seroma fluid, respectively.

According to the serogroup determination, the clinical isolates belonged to serogroups IVb (83% of the isolates) and IIa (17% of the isolates). All of the clinical strains belonging to serogroup IVb were isolated in subjects at risk of listeriosis (namely newborns, pregnant women, the elderly, and cancer patients with meningitis). Conversely, only two out of the seven clinical strains belonging to serogroup IIa were isolated in at-risk patients (namely two elderly people with ulcerative colitis and sepsis and with subcutaneous fluid, respectively).

**Figure 2:**
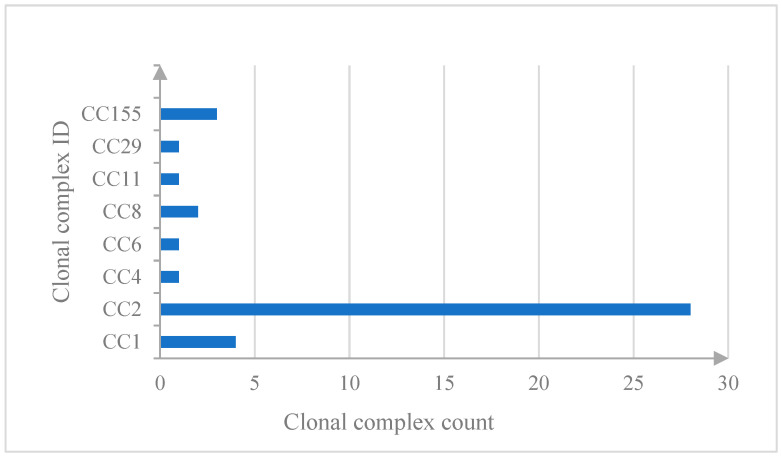
Clonal complex identified between 2019 and 2023 in Sicily.

Overall, eight MLST CCs were identified; in particular, CC2/ST2 (28/41, 68.3%), CC1/ST1(4/41, 9.7%), CC155/ST155 (3/41, 7.3%), CC8/ST8 (2/41, 4.9%), CC11/ST451, CC29/ST29, CC4/ST219, and CC6/ST6 (1/41, 2.4% each). The data are shown in Figure 2. According to the literature [33], CC2/ST2 specimens are strongly associated with clinical origins, in particular with the central nervous system (CNS) or maternal–neonatal (MN) listeriosis cases.

All 41 isolates were found to be susceptible to ampicillin, erythromycin, and meropenem; 12/41 strains (29.23%) were resistant to trimethoprim/sulfamethoxazole; and 1/41 (2.3%) were resistant to penicillin G. The resistant strains belonged to serogroup IVb.

The cgMLST was performed on a total number of 63 CC2-ST2 strains. Based on the aforementioned analyses, the n.1 CC2 strain from food belonging to ST67 and n.5 strains from clinical cases, having vertical coverage of <30 and having shown AD > 7 based on previous clustering performed using the IRIDA-ARIES platform (n = 5), were excluded. The details are reported in Supplementary Table S1.

**Figure 3:**
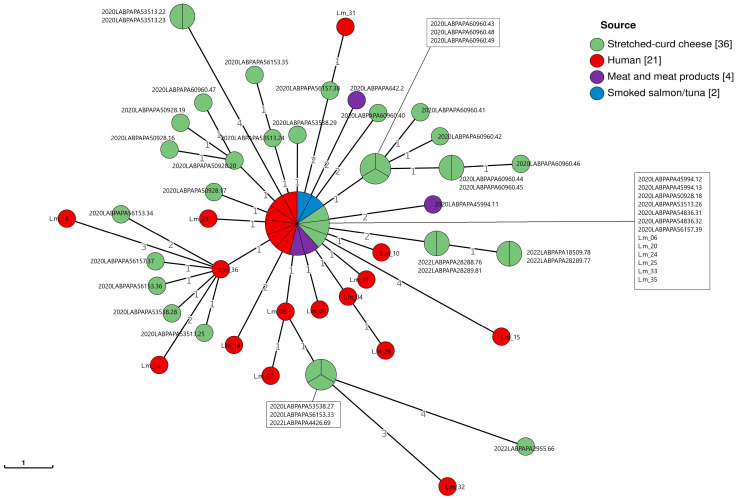
Minimum Spanning Tree (MST) based on the cgMLST profiles of *L. monocytogenes* CC2-ST2, colored according to the source of the strains. The MST was visualized using GrapeTree (https://github.com/achtman-lab/GrapeTree (accessed on 28 February 2025)). The numbers in square brackets correspond to the number of samples belonging to each source category.

**Figure 4:**
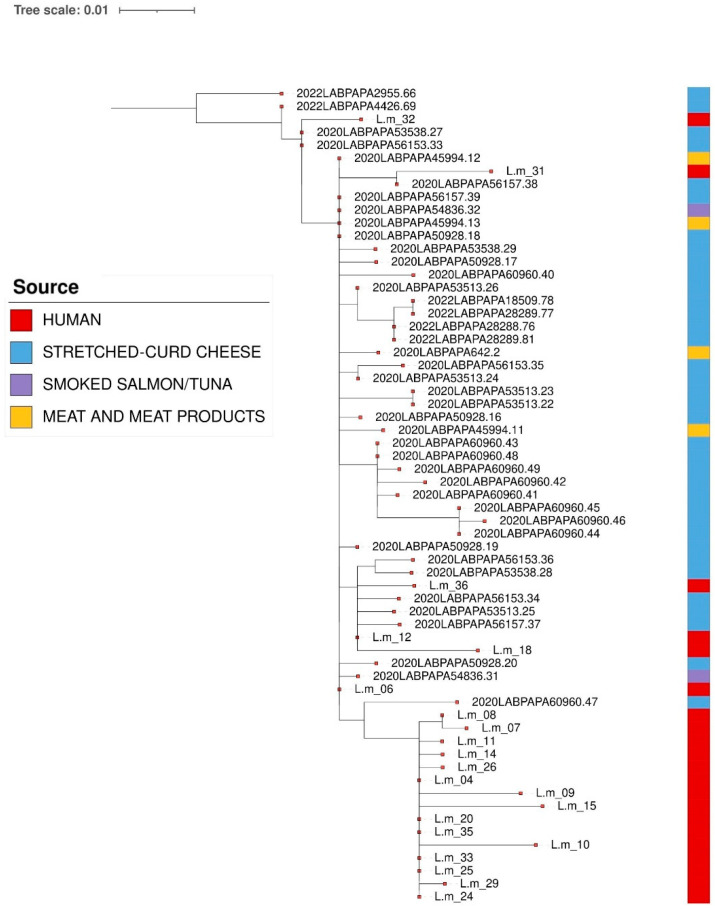
Maximum Likelihood (ML) tree obtained from CFSAN pipeline of *L. monocytogenes* CC2-ST2. The first layer represents the source of isolation, as shown in the legend. The tree was visualized using the Interactive Tree of Life (iTOL) https://itol.embl.de/ (accessed on 28 February 2025).

According to the cgMLST, one cluster was detected among *L. monocytogenes* CC2-ST2 strains isolated from both clinical samples and foods, mainly from the stretched-curd cheese samples (Figure 3). This result was confirmed via SNP analysis, as reported in Figure 4.

**Figure 5:**
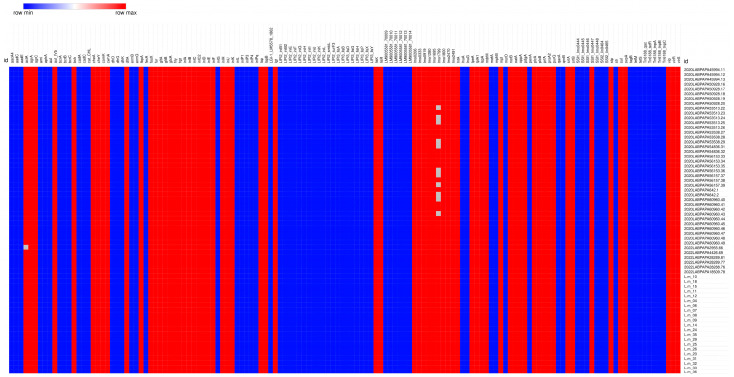
Heatmap reporting the presence/absence of *L. monocytogenes* CC2-ST2 virulence and resistance profiles. Red square: presence of the gene. Blue square: absence of the gene. White square: truncated gene. The heatmap was generated using Morpheus https://software.broadinstitute.org/morpheus (accessed on 28 February 2025).

The results related to the presence or absence of virulence, antibiotic, and stress resistance genes in the *L. monocytogenes* CC2-ST2 strains are reported in Figure 5.

All of the *L. monocytogenes* CC2-ST2 strains showed the same virulence and resistance profile, except for some isolates from food, carrying truncated genes such as *actA*, one of the six genes belonging to the *L. monocytogenes* Pathogenicity Island (LIPI) 1, or *lmo1799*, a gene involved in stress resistance [34,35].

Regarding the virulence profile, the *L. monocytogenes* CC2-ST2 strains had a complete LIPI-1, involved in the intracellular infection cycle of *L. monocytogenes;* a full-length *inlA* and a full-length *inlB*, sequences encoding Internalin A and Internalin B, respectfully, which are both crucial for the *L. monocytogenes* invasion of host cells [34]. No other accessory LIPIs, such as LIPI-2, LIPI-3, and LIPI-4 [33,34,36] were found in the isolates considered.

With regard to resistance to stresses, none of the strains tested carried *cadA* genes and transposons of the *TN3* family [37,38] or genes of the *bcrABC* cassette [39], known to be related to metal and disinfectant resistance, respectively. On the contrary, all of the aforementioned isolates carried an incomplete *Stress Survival Islet* 1 (SSI-1), known to contribute to the survival of *L. monocytogenes* in food environments, under stressful conditions and here characterized by the presence of only one out of the five genes composing the SSI-1 (*lmo0447*) [40].

Finally, referring to Antimicrobial Resistance Genes (ARGs), all the strains tested carried intrinsic core genes, in particular, *fosX* (resistance to fosfomycin), *norB* (resistance to quinolones) [21], *sul* (resistance to sulfonamides), and *lmo0919* (resistance to lincosamides) [41,42].

## 4. Discussion

In this work, we reported the data from food control, the epidemiological data recorded on a voluntary basis, and the data from surveillance of clinical cases, collected in Sicily between 2019 and 2023.

The molecular characterization of isolates revealed the presence of two predominant serogroups, namely IVb (69% among food-source isolates and 90.2% of the clinical isolates) and IIa (28.7% among food-source isolates and 9.7% of the clinical isolates). These results are in line with national [43] and European [44] data and agree with references reporting serogroups IIa, IIb, and IVb as responsible for the majority of cases of listeriosis, and in particular, serogroups IVb and, to a lesser extent, IIa as the ones more frequently associated with epidemic outbreaks [45]. Moreover, a higher number of cases of listeriosis was recorded in patients aged over 65, coinciding with the trend data published in the last European Union One Health Zoonoses report [5]. Specifically referring to food-source isolates, the predominance recorded for serogroup IVb and reported in this study is also partially due to the presence, among dairy products, of samples collected from the same producing plant, both as finished products and along the production chain, in 2020 and 2022. Hence, these samples were contaminated by one single strain (CC2, ST2), capable of persisting for 3 years on the surface of machineries and/or worktops, despite the eradication measures implemented after its first detection.

The resilience and ability to colonize and persist within food processing facilities are well-known qualities of *L. monocytogenes* [21,46] as well as the ability to form biofilms and proliferate not only on surfaces but also in harsh environments despite the application of sanitization practices [47]. For this reason, the transmission of *L. monocytogenes* can be mediated by multiple food classes, including RTE delicatessen, raw, smoked, and frozen fish, dairy products, meat products, and vegetables [21,46,48,49,50].

Although in the present study, a greater variability among the strains isolated was observed, referring to meat and meat products and fish products in terms of both serogroup and CC and ST, the majority of strains belonged to CC2-ST2, found in all food types except for vegetables. The absence of CC2-ST2 strains among vegetable-sourced isolates, however, could also be linked to the small sample size of this group, accounting for 6.8% of the total strains analyzed in this study, and this does not exclude its potential of also being transmitted by vegetables. In fact, during a study period overlapping with ours, CC2-ST2 isolates were also detected in processing water from FNAO (Food of Non-Animal Origin) producing/processing plants in Germany [51].

The clustering analysis with both cgMLST and SNPs methods highlighted the presence of a CC2-ST2 cluster involving strains isolated from food and human samples characterized by genomic correlation, showing an AD ≤ 7 and a SNP difference of ≤10 [52]. Nevertheless, it is necessary to underline that genomic correlation does not represent sufficient evidence to indicate a causal connection for disease between strains (human and food matrices). Indeed, the link must be necessarily confirmed or excluded based on epidemiological evidence. In addition, the WGS analyses underline that, even if the majority of strains clustering with the clinical strains belonged to the stretched-curd cheese, more than one food matrix, belonging to different food categories, showed genomic correlation.

CC2 is well known for being a hypervirulent clone, often associated with clinical infections [33]. A predominance of CC2-ST2 was also recorded among clinical isolates and is in agreement with the literature. In fact, according to Italian data, CC-ST2 was the most frequent sequence type connected to Sicilian listeriosis. Since 1997, ST2 has been associated with the largest epidemic outbreak in Italy, with it being responsible for the hospitalization of 292 people (20% of patients) (children and primary school staff), caused by eating contaminated tuna and corn salad [32]. A clear connection of specific serotypes to certain foods cannot be made, but according to the literature, a marked association with meat foods of serotype IIc, and in particular of sequence type ST9, is evident, although its predominance over other serotypes and subtypes cannot be affirmed [21,53,54]. In line with the literature, the present study detected serotype IIc and sequence type ST9 solely among meat products, even with no predominance over the other types and subtypes revealed. Referring to the assessment of antimicrobial resistance, the majority of strains were susceptible to most of the tested antibacterial agents, including those recommended as first-line and second-line choices for the treatment of severe listeriosis in humans, i.e., ampicillin, penicillin G (only 2.3% of clinical isolates and 7.7% of food-source isolates were resistant), gentamicin, tetracycline, erythromycin, vancomycin, and chloramphenicol. These data agree with results recently obtained by others, reporting sensitivity to the majority of antimicrobials recommended as first-line and alternative choices for the treatment of severe listeriosis [45,55]. Despite a general susceptibility profile of the strains of *L. monocytogenes* analyzed, resistance to trimethoprim/sulfamethoxazole (29.23% of clinical strains), oxacillin (85.9% of food-source isolates), as well as intermediate resistance to clindamycin (34.6% of food-source isolates) were recorded. These results are worthy of attention and underline the need for timely monitoring, especially considering that relevant resistance rates to the aforementioned antimicrobials were already reported, although with different percentage values [20,32,45]. Even though a low percentage of multi-drug-resistant (MDR) strains was detected among isolates of food origin (7.7%), this result is worthy of attention. Further analysis should be performed to assess any increase in this figure, especially considering that several studies report higher percentages of MDR strains in food, food-processing plants, and human samples [45,47,56,57]. With regard to the presence/absence of virulence and antibiotic- and stress-resistance genes in the studied CC2-ST2 strains, no differences were revealed between the clinical and food-source isolates. Equally, the antimicrobial resistance pattern, obtained via WGS, solely included the intrinsic core genes, hence confirming that the acquisition of ARGs through horizontal gene transfer is uncommon in *L. monocytogenes*, as reported by Moura et al. [55]. Indeed, references on this topic are controversial and make this topic worthy of further investigation. In fact, even though *L. monocytogenes* is generally known for unfrequently acquiring exogenous genes through horizontal gene transfer [55,58], ARGs encoded on mobile genetic elements have been reported in various isolates of *L. monocytogenes*, both from foods and clinical samples [47,59,60,61,62].

Similar to other studies, the antimicrobial resistance phenotype recorded in *L. monocytogenes* isolates did not always associate with the corresponding ARGs encoded by mobile genetic elements. These data suggest that the antibiotic resistance phenotypes revealed could be attributed to other mechanisms, such as reduced permeability, efflux pump activation, or chromosomal mutations, especially referring to MDR isolates [47,62].

Overall, our results highlight the persistence and circulation of some CCs among various food matrices and clinical specimens in Sicily during the period under study, especially CC2. The presence of this well-known hypervirulent clone in foods could be a crucial concern for consumer safety. Scientific results obtained during this five-year period showed that, in the future, the information related to strain characterization and epidemiological data should be shared among the various bodies and competent authorities participating in the surveillance of listeriosis in real time at all levels (local, regional, and national) in order to evaluate the possible correlations among the isolated strains. Considering the marked resilience of *L. monocytogenes* and the emergence of resistance profiles that could affect the outcome of therapies for human listeriosis, the need to consolidate an integrated and punctual monitoring system for both human and environment health is evident.

## Figures and Tables

**Table 1:** Target gene and size expected for the specific amplicons.

Determination	Target Gene	Amplicon Size	References
Species *L. monocytogenes*	*prfA*	274 bp	[24,25]
Serogroup	*lmo0737*	691 bp	[26]
Serogroup	*lmo1118*	906 bp	[26]
Serogroup	*ORF2819*	471 bp	[26]
Serogroup	*ORF2110*	597 bp	[26]

**Table 2:** Details related to *L. monocytogenes* isolates from food matrices.

Type of Food		No. of Isolates	Year	Serogroup	Clonal Complex (CC)	Sequence Type (ST)
Dairy products	Ricotta cheese/ ricotta cream	5 ^1^	2020	IIa	CC199	ST199
		2 ^1^	2020	IVb	CC6	ST6
	Stretched-curd cheese	31 ^2^	2020	IVb	CC2	ST2
		3 ^2^	2022	IVb	CC2	ST2
		2	2022	IVb	CC2	ST2
		1	2022	IIa	CC204	ST204
Meat and meat products		1	2020	IVb	CC2	ST67
		4	2020	IVb	CC2	ST2
		1	2020	IIa	CC14	ST14
		2	2020	IIa	CC155	ST155
		1	2020	IVb	CC6	ST6
		1	2021	IIc	CC9	ST9
		2	2021	IVb	CC1	ST1
		2	2021	IIa	CC475	ST504
		1	2022	IIc	CC9	ST763
		2	2022	IIa	CC121	ST121
		1	2022	IIa	CC14	ST399
		3	2022	IIa	CC8	ST8
Fish products	seafood salad	3	2020	IIa	CC8	ST8
		1	2021			
		1	2020	IIb	CC426	ST426
		2	2022			
		1	2023			
		1	2022	IIa	CC121	ST121
	smoked salmon/ tuna	2	2020	IIb	CC3	ST3
		1	2020	IIa	CC7	ST7
		1	2021			
		2	2020	IVb	CC2	ST2
		1	2022	IIb	CC87	ST87
		1	2022	IIa	CC155	ST155
Vegetables		3	2020	IVb	CC1	ST1
		1	2023			
		1	2022	IVb	CC6	ST6

^1^ Strains isolated from samples from the same dairy. ^2^ Strains isolated from samples collected at the same producing plant.

## Data Availability

The original contributions presented in this study are included in the article. Further inquiries can be directed to the corresponding author.

## Supplementary Materials

The following supporting information can be downloaded at: https://www.mdpi.com/article/10.3390/pathogens14030294/s1, Table S1: Strain IDs from Figure 3, Figure 4 and Figure 5 and corresponding source sample.

## References

[1] Amagliani G., Blasi G., Scuota S., Duranti A., Fisichella S., Gattuso A., Gianfranceschi M.V., Schiavano G.F., Brandi G., Pomilio F. (2021). Detection and Virulence Characterization of *Listeria monocytogenes* Strains in Ready-to-Eat Products. Foodborne Pathog. Dis..

[2] Ferreira V., Wiedmann M., Teixeira P., Stasiewicz M.J. (2014). *Listeria monocytogenes* Persistence in Food-Associated Environments: Epidemiology, Strain Characteristics, and Implications for Public Health. J. Food Prot..

[3] Ricci A., Allende A., Bolton D., Chemaly M., Davies R., Fernandez-Escamez P.S., Girones R., Herman L., Koutsoumanis K., EFSA BIOHAZ Panel (EFSA Panel on Biological Hazards) (2018). Scientific Opinion on the *Listeria monocytogenes* Contamination of Ready-to-Eat Foods and the Risk for Human Health in the EU. EFSA J..

[4] Lundén J., Autio T., Markkula A., Hellström S., Korkeala H. (2003). Adaptive and Cross-Adaptive Responses of Persistent and Non-Persistent *Listeria monocytogenes* Strains to Disinfectants. Int. J. Food Microbiol..

[5] European Food Safety Authority (EFSA), European Centre for Disease Prevention and Control (ECDC) (2024). The European Union One Health 2023 Zoonoses Report. EFS2.

[6] European Centre for Disease Prevention and Control (2024). Threats and Outbreaks of Listeriosis. https://www.ecdc.europa.eu/en/listeriosis/threats-and-outbreaks.

[7] Eurostat (European Commission) Population Structure and Ageing. https://ec.europa.eu/eurostat/statistics-explained/index.php?title=population_structure_and_ageing.

[8] Koutsoumanis K., Alvarez-Ordóñez A., Bolton D., Bover-Cid S., Chemaly M., Davies R., De Cesare A., Herman L., Hilbert F., EFSA BIOHAZ Panel (EFSA Panel on Biological Hazards) (2020). The Public Health Risk Posed by *Listeria monocytogenes* in Frozen Fruit and Vegetables Including Herbs, Blanched during Processing. EFSA J..

[9] Quereda J.J., Morón-García A., Palacios-Gorba C., Dessaux C., García-del Portillo F., Pucciarelli M.G., Ortega A.D. (2021). Pathogenicity and Virulence of *Listeria monocytogenes*: A Trip from Environmental to Medical Microbiology. Virulence.

[10] Weindl L., Frank E., Ullrich U., Heurich M., Kleta S., Ellerbroek L., Gareis M. (2016). *Listeria monocytogenes* in Different Specimens from Healthy Red Deer and Wild Boars. Foodborne Pathog. Dis..

[11] Hellström S., Kiviniemi K., Autio T., Korkeala H. (2008). *Listeria monocytogenes* Is Common in Wild Birds in Helsinki Region and Genotypes Are Frequently Similar with Those Found along the Food Chain. J. Appl. Microbiol..

[12] Gu Y., Liang X., Huang Z., Yang Y. (2015). Outbreak of *Listeria monocytogenes* in Pheasants. Poult. Sci..

[13] Cao J., Zhang J., Ma L., Li L., Zhang W., Li J. (2018). Identification of Fish Source *Vibrio alginolyticus* and Evaluation of Its Bacterial Ghosts Vaccine Immune Effects. MicrobiologyOpen.

[14] Iwu C.D., Okoh A.I. (2020). Characterization of Antibiogram Fingerprints in *Listeria monocytogenes* Recovered from Irrigation Water and Agricultural Soil Samples. PLoS ONE.

[15] Schoder D., Schmalwieser A., Szakmary-Brändle K., Stessl B., Wagner M. (2015). Urban Prevalence of *Listeria* spp. and *Listeria monocytogenes* in Public Lavatories and on Shoe Soles of Facility Patrons in the European Capital City Vienna. Zoonoses Public Health.

[16] European Food Safety Authority (EFSA), European Centre for Disease Prevention and Control (ECDC) (2022). Collaboration Agreement on the Management and Sharing of Molecular Typing Data of Isolates from Human, Food, Feed, Animal, and the Related En-Vironment for Public Health Purposes. https://www.efsa.europa.eu/sites/default/files/2022-06/collaboration-agreement-molecular-typing-EFSA-ECDC-WGS-DataCollection.pdf.

[17] (2017). Microbiology of the Food Chain—Horizontal Method for the Detection and Enumeration of *Listeria monocytogenes* and of *Listeria* spp.—Part 1: Detection Method.

[18] European Committee on Antimicrobial Susceptibility Testing (EUCAST). https://www.eucast.org/.

[19] CLSI (2021). Performance Standards for Antimicrobial Susceptibility: Supplement M100.

[20] Andriyanov P.A., Zhurilov P.A., Liskova E.A., Karpova T.I., Sokolova E.V., Yushina Y.K., Zaiko E.V., Bataeva D.S., Voronina O.L., Psareva E.K. (2021). Antimicrobial Resistance of *Listeria monocytogenes* Strains Isolated from Humans, Animals, and Food Products in Russia in 1950–1980, 2000–2005, and 2018–2021. Antibiotics.

[21] Silva A., Silva V., Gomes J.P., Coelho A., Batista R., Saraiva C., Esteves A., Martins Â., Contente D., Diaz-Formoso L. (2024). *Listeria monocytogenes* from Food Products and Food Associated Environments: Antimicrobial Resistance, Genetic Clustering and Biofilm Insights. Antibiotics.

[22] Torresi M., Ruolo A., Acciari V.A., Ancora M., Blasi G., Cammà C., Centorame P., Centorotola G., Curini V., Guidi F. (2020). A Real-Time PCR Screening Assay for Rapid Detection of *Listeria monocytogenes* Outbreak Strains. Foods.

[23] Chiarini A., Palmeri A., Amato T., Immordino R., Distefano S., Giammanco A. (2008). Detection of Bacterial and Yeast Species with the Bactec 9120 Automated System with Routine Use of Aerobic, Anaerobic, and Fungal Media. J. Clin. Microbiol..

[24] D’agostino M., Wagner M., Vazquez-Boland J.A., Kuchta T., Karpiskova R., Hoorfar J., Novella S., Scortti M., Ellison J., Murray A. (2004). A Validated PCR-Based Method to Detect *Listeria monocytogenes* Using Raw Milk as a Food Model—Towards an International Standard. J. Food Prot..

[25] Kérouanton A., Marault M., Petit L., Grout J., Dao T.T., Brisabois A. (2010). Evaluation of a Multiplex PCR Assay as an Alternative Method for *Listeria monocytogenes* Serotyping. J. Microbiol. Methods.

[26] Doumith M., Jacquet C., Gerner-Smidt P., Graves L.M., Loncarevic S., Mathisen T., Morvan A., Salcedo C., Torpdahl M., Vazquez J.A. (2005). Multicenter Validation of a Multiplex PCR Assay for Differentiating the Major *Listeria monocytogenes* Serovars 1/2a, 1/2b, 1/2c, and 4b: Toward an International Standard. J. Food Prot..

[27] Centorotola G., Ziba M.W., Cornacchia A., Chiaverini A., Torresi M., Guidi F., Cammà C., Bowa B., Mtonga S., Magambwa P. (2023). *Listeria monocytogenes* in Ready to Eat Meat Products from Zambia: Phenotypical and Genomic Characterization of Isolates. Front. Microbiol..

[28] (2022). Microbiology of the Food Chain-Whole Genome Sequencing for Typing and Genomic Characterization of Bacteria-General Requirements and Guidance.

[29] Moura A., Criscuolo A., Pouseele H., Maury M.M., Leclercq A., Tarr C., Björkman J.T., Dallman T., Reimer A., Enouf V. (2016). Whole Genome-Based Population Biology and Epidemiological Surveillance of *Listeria monocytogenes*. Nat. Microbiol..

[30] Zhou Z., Alikhan N.-F., Sergeant M.J., Luhmann N., Vaz C., Francisco A.P., Carriço J.A., Achtman M. (2018). GrapeTree: Visualization of Core Genomic Relationships among 100,000 Bacterial Pathogens. Genome Res..

[31] Davis S., Pettengill J.B., Luo Y., Payne J., Shpuntoff A., Rand H., Strain E. (2015). CFSAN SNP Pipeline: An Automated Method for Constructing SNP Matrices from next-Generation Sequence Data. PeerJ Comput. Sci..

[32] Tricoli M.R., Massaro C., Arrigo I., Diquattro O., Di Bernardo F., Galia E., Palermo M., Fasciana T., Giammanco A. (2024). Characterization of *Listeria monocytogenes* Strains Isolated in Palermo (Sicily and Italy) during the Years 2018–2020 from Severe Cases of Listeriosis. Antibiotics.

[33] Maury M.M., Tsai Y.-H., Charlier C., Touchon M., Chenal-Francisque V., Leclercq A., Criscuolo A., Gaultier C., Roussel S., Brisabois A. (2016). Uncovering *Listeria monocytogenes* Hypervirulence by Harnessing Its Biodiversity. Nat. Genet..

[34] Vázquez-Boland J.A., Kuhn M., Berche P., Chakraborty T., Domínguez-Bernal G., Goebel W., González-Zorn B., Wehland J., Kreft J. (2001). *Listeria* Pathogenesis and Molecular Virulence Determinants. Clin. Microbiol. Rev..

[35] Avila-Novoa M.G., González-Torres B., González-Gómez J.P., Guerrero-Medina P.J., Martínez-Chávez L., Martínez-Gonzáles N.E., Chaidez C., Gutiérrez-Lomelí M. (2023). Genomic Insights into *Listeria monocytogenes*: Organic Acid Interventions for Biofilm Prevention and Control. Int. J. Mol. Sci..

[36] Lee S., Parsons C., Chen Y., Dungan R.S., Kathariou S. (2023). Contrasting Genetic Diversity of *Listeria* Pathogenicity Islands 3 and 4 Harbored by Nonpathogenic *Listeria* spp. Appl. Environ. Microbiol..

[37] Parsons C., Lee S., Jayeola V., Kathariou S. (2017). Novel Cadmium Resistance Determinant in *Listeria monocytogenes*. Appl. Environ. Microbiol..

[38] Lebrun M., Audurier A., Cossart P. (1994). Plasmid-Borne Cadmium Resistance Genes in *Listeria monocytogenes* Are Present on Tn5422, a Novel Transposon Closely Related to Tn917. J. Bacteriol..

[39] Dutta V., Elhanafi D., Kathariou S. (2013). Conservation and Distribution of the Benzalkonium Chloride Resistance Cassette *bcrABC* in *Listeria monocytogenes*. Appl. Environ. Microbiol..

[40] Ryan S., Begley M., Hill C., Gahan C.G.M. (2010). A Five-Gene Stress Survival Islet (SSI-1) That Contributes to the Growth of *Listeria monocytogenes* in Suboptimal Conditions: Stress Survival Islet in *L. monocytogenes*. J. Appl. Microbiol..

[41] Goh Y.-X., Anupoju S.M.B., Nguyen A., Zhang H., Ponder M., Krometis L.-A., Pruden A., Liao J. (2024). Evidence of Horizontal Gene Transfer and Environmental Selection Impacting Antibiotic Resistance Evolution in Soil-Dwelling *Listeria*. Nat. Commun..

[42] Chesneau O., Ligeret H., Hosan-Aghaie N., Morvan A., Dassa E. (2005). Molecular Analysis of Resistance to Streptogramin A Compounds Conferred by the Vga Proteins of *Staphylococci*. Antimicrob. Agents Chemother..

[43] https://www.epicentro.iss.it/listeria/.

[44] https://atlas.ecdc.europa.eu/public/index.aspx.

[45] Lachtara B., Wieczorek K., Osek J. (2023). Antimicrobial Resistance of *Listeria monocytogenes* Serogroups IIa and IVb from Food and Food-Production Environments in Poland. J. Vet. Res..

[46] Tirloni E., Centorotola G., Pomilio F., Torresi M., Bernardi C., Stella S. (2024). *Listeria monocytogenes* in Ready-to-Eat (RTE) Delicatessen Foods: Prevalence, Genomic Characterization of Isolates and Growth Potential. Int. J. Food Microbiol..

[47] Kayode A.J., Okoh A.I. (2022). Assessment of Multidrug-Resistant *Listeria monocytogenes* in Milk and Milk Product and One Health Perspective. PLoS ONE.

[48] Dos Reis J.O., Vieira B.S., Cunha Neto A., Castro V.S., Figueiredo E.E.D.S. (2022). Antimicrobial Resistance of *Listeria monocytogenes* from Animal Foods to First- and Second-Line Drugs in the Treatment of *Listeriosis* from 2008 to 2021: A Systematic Review and Meta-Analysis. Can. J. Infect. Dis. Med. Microbiol..

[49] Truong H., Garmyn D., Gal L., Fournier C., Sevellec Y., Jeandroz S., Piveteau P. (2021). Plants as a Realized Niche for *Listeria monocytogenes*. Microbiol. Open.

[50] Ramos B., Brandão T.R.S., Teixeira P., Silva C.L.M. (2020). Biopreservation Approaches to Reduce *Listeria monocytogenes* in Fresh Vegetables. Food Microbiol..

[51] Wartha S., Bretschneider N., Dangel A., Hobmaier B., Hörmansdorfer S., Huber I., Murr L., Pavlovic M., Sprenger A., Wenning M. (2023). Genetic Characterization of *Listeria* from Food of Non-Animal Origin Products and from Producing and Processing Companies in Bavaria, Germany. Foods.

[52] Segerman B., Skarin H., Ástvaldsson Á. (2024). Guidance Document for Cluster Analysis of Whole Genome Sequence Data.

[53] Alvarez-Molina A., Cobo-Díaz J.F., López M., Prieto M., De Toro M., Alvarez-Ordóñez A. (2021). Unraveling the Emergence and Population Diversity of *Listeria monocytogenes* in a Newly Built Meat Facility through Whole Genome Sequencing. Int. J. Food Microbiol..

[54] Pérez-Baltar A., Pérez-Boto D., Medina M., Montiel R. (2021). Genomic Diversity and Characterization of *Listeria monocytogenes* from Dry-Cured Ham Processing Plants. Food Microbiol..

[55] Moura A., Leclercq A., Vales G., Tessaud-Rita N., Bracq-Dieye H., Thouvenot P., Madec Y., Charlier C., Lecuit M. (2024). Phenotypic and Genotypic Antimicrobial Resistance of *Listeria monocytogenes*: An Observational Study in France. Lancet Reg. Health—Eur..

[56] Wiśniewski P., Zakrzewski A.J., Zadernowska A., Chajęcka-Wierzchowska W. (2022). Antimicrobial Resistance and Virulence Characterization of *Listeria monocytogenes* Strains Isolated from Food and Food Processing Environments. Pathogens.

[57] Noll M., Kleta S., Al Dahouk S. (2018). Antibiotic Susceptibility of 259 *Listeria monocytogenes* Strains Isolated from Food, Food-Processing Plants and Human Samples in Germany. J. Infect. Public Health.

[58] Baquero F., Lanza V.F., Duval M., Coque T.M. (2020). Ecogenetics of Antibiotic Resistance in *Listeria monocytogenes*. Mol. Microbiol..

[59] Allen K.J., Wałecka-Zacharska E., Chen J.C., Katarzyna K.-P., Devlieghere F., Van Meervenne E., Osek J., Wieczorek K., Bania J. (2016). *Listeria monocytogenes*—An Examination of Food Chain Factors Potentially Contributing to Antimicrobial Resistance. Food Microbiol..

[60] Srinivasan V., Nam H.M., Nguyen L.T., Tamilselvam B., Murinda S.E., Oliver S.P. (2005). Prevalence of Antimicrobial Resistance Genes in *Listeria monocytogenes* Isolated from Dairy Farms. Foodborne Pathog. Dis..

[61] Morvan A., Moubareck C., Leclercq A., Hervé-Bazin M., Bremont S., Lecuit M., Courvalin P., Le Monnier A. (2010). Antimicrobial Resistance of *Listeria monocytogenes* Strains Isolated from Humans in France. Antimicrob. Agents Chemother..

[62] Heidarzadeh S., Pourmand M.R., Hasanvand S., Pirjani R., Afshar D., Noori M., Soltan Dallal M.M. (2021). Antimicrobial Susceptibility, Serotyping, and Molecular Characterization of Antibiotic Resistance Genes in *Listeria monocytogenes* Isolated from Pregnant Women with a History of Abortion. Iran. J. Public Health.

