# Supplementary material for: Molecular Characterization and Antimicrobial Resistance Evaluation of Listeria monocytogenes Strains from Food and Human Samples
Source: Pathogens. 2025 Mar 18;14(3):294. doi: 10.3390/pathogens14030294 (PMC11945527; doi:10.3390/pathogens14030294)
Supplement: Supplementary file 1 [file pathogens-14-00294-s001.zip › pathogens-3486063-supplementary.pdf]

Table S1. Strain IDs from Figures 3-5 and corresponding source sample and accession number.

n.	Strain Identification	Clonal complex (CC)	Sequence type (ST)	Source	BioSample accession number
1	2020LABPAPA642.2	CC2	2	Meat and meat products	SAMN47300141
2	2020LABPAPA45994.11	CC2	2	Meat and meat products	SAMN47300142
3	2020LABPAPA45994.12	CC2	2	Meat and meat products	SAMN47300143
4	2020LABPAPA45994.13	CC2	2	Meat and meat products	SAMN47300144
5	2020LABPAPA50928.16	CC2	2	Stretched-curd cheese	SAMN47300145
6	2020LABPAPA50928.17	CC2	2	Stretched-curd cheese	SAMN47300146
7	2020LABPAPA50928.18	CC2	2	Stretched-curd cheese	SAMN47300147
8	2020LABPAPA50928.19	CC2	2	Stretched-curd cheese	SAMN47300148
9	2020LABPAPA50928.20	CC2	2	Stretched-curd cheese	SAMN47300149
10	2020LABPAPA53513.22	CC2	2	Stretched-curd cheese	SAMN47300150
11	2020LABPAPA53513.23	CC2	2	Stretched-curd cheese	SAMN47300151
12	2020LABPAPA53513.24	CC2	2	Stretched-curd cheese	SAMN47300152
13	2020LABPAPA53513.25	CC2	2	Stretched-curd cheese	SAMN47300153
14	2020LABPAPA53513.26	CC2	2	Stretched-curd cheese	SAMN47300154
15	2020LABPAPA53538.27	CC2	2	Stretched-curd cheese	SAMN47300155
16	2020LABPAPA53538.28	CC2	2	Stretched-curd cheese	SAMN47300156
17	2020LABPAPA53538.29	CC2	2	Stretched-curd cheese	SAMN47300157
18	2020LABPAPA54836.31	CC2	2	Smoked salmon/tuna	SAMN47300158
19	2020LABPAPA54836.32	CC2	2	Smoked salmon/tuna	SAMN47300159
20	2020LABPAPA56153.33	CC2	2	Stretched-curd cheese	SAMN47300160
21	2020LABPAPA56153.34	CC2	2	Stretched-curd cheese	SAMN47300161
22	2020LABPAPA56153.35	CC2	2	Stretched-curd cheese	SAMN47300162
23	2020LABPAPA56153.36	CC2	2	Stretched-curd cheese	SAMN47300163
24	2020LABPAPA56157.37	CC2	2	Stretched-curd cheese	SAMN47300164
25	2020LABPAPA56157.38	CC2	2	Stretched-curd cheese	SAMN47300165
26	2020LABPAPA56157.39	CC2	2	Stretched-curd cheese	SAMN47300166
27	2020LABPAPA60960.40	CC2	2	Stretched-curd cheese	SAMN47300167
28	2020LABPAPA60960.41	CC2	2	Stretched-curd cheese	SAMN47300168
29	2020LABPAPA60960.42	CC2	2	Stretched-curd cheese	SAMN47300169
30	2020LABPAPA60960.43	CC2	2	Stretched-curd cheese	SAMN47300170
31	2020LABPAPA60960.44	CC2	2	Stretched-curd cheese	SAMN47300171
32	2020LABPAPA60960.45	CC2	2	Stretched-curd cheese	SAMN47300172
33	2020LABPAPA60960.46	CC2	2	Stretched-curd cheese	SAMN47300173
34	2020LABPAPA60960.47	CC2	2	Stretched-curd cheese	SAMN47300174
35	2020LABPAPA60960.48	CC2	2	Stretched-curd cheese	SAMN47300175
36	2020LABPAPA60960.49	CC2	2	Stretched-curd cheese	SAMN47300176
37	2022LABPAPA2955.66	CC2	2	Stretched-curd cheese	SAMN47300177
38	2022LABPAPA4426.69	CC2	2	Stretched-curd cheese	SAMN47300178
39	2022LABPAPA28288.76	CC2	2	Stretched-curd cheese	SAMN47300179
40	2022LABPAPA28289.77	CC2	2	Stretched-curd cheese	SAMN47300180
41	2022LABPAPA18509.78	CC2	2	Stretched-curd cheese	SAMN47300181
42	2022LABPAPA28289.81	CC2	2	Stretched-curd cheese	SAMN47300182
43	L.m_04	CC2	2	Clinical	SAMN47300183
44	L.m_06	CC2	2	Clinical	SAMN47300184

45	L.m_07	CC2	2	Clinical	SAMN47300185
46	L.m_08	CC2	2	Clinical	SAMN47300186
47	L.m_09	CC2	2	Clinical	SAMN47300187
48	L.m_20	CC2	2	Clinical	SAMN47300188
49	L.m_32	CC2	2	Clinical	SAMN47300189
50	L.m_33	CC2	2	Clinical	SAMN47300190
51	L.m_29	CC2	2	Clinical	SAMN47300191
52	L.m_26	CC2	2	Clinical	SAMN47300192
53	L.m_25	CC2	2	Clinical	SAMN47300193
54	L.m_24	CC2	2	Clinical	SAMN47300194
55	L.m_18	CC2	2	Clinical	SAMN47300195
56	L.m_14	CC2	2	Clinical	SAMN47300196
57	L.m_15	CC2	2	Clinical	SAMN47300197
58	L.m_12	CC2	2	Clinical	SAMN47300198
59	L.m_10	CC2	2	Clinical	SAMN47300199
60	L.m_11	CC2	2	Clinical	SAMN47300200
61	L.m_36	CC2	2	Clinical	SAMN47300201
62	L.m_35	CC2	2	Clinical	SAMN47300202
63	L.m_31	CC2	2	Clinical	SAMN47300203
